# Weight gain as a secondary effect of Olanzapine, and its interference in treatment adherence: a case report.

**DOI:** 10.1192/j.eurpsy.2023.2153

**Published:** 2023-07-19

**Authors:** P. Setién Preciados, E. Arroyo Sánchez

**Affiliations:** Psychiatry, Hospital Universitario Príncipe de Asturias, Alcalá de Henares, Spain

## Abstract

**Introduction:**

Olanzapine is a second generation antipsychotic that is approved for the treatment of schizophrenia, bipolar disorder type 1 as monotherapy, or as an add-on to lithium or valproate (manic or mixed episodes), and it is also used off label for acute anxiety, insomnia… It is one of the most effective antipsychotics but concerns remain due to its significant metabolic adverse effects. Olanzapine has one of the highest rates of weight gain among all antipsychotic drugs, which challenges patient’s adherence to treatment.

**Objectives:**

Review how much influence Olanzapine has on weight gain, its influence in treatment adherence and alternatives in clinical practice.

**Methods:**

Presentation of a patient’s case and review of existing literature, in regards to Olanzapine and its repercussions on weight gain and the old and new alternatives available right now.

**Results:**

Olanzapine is an effective antipsychotic, however, it causes secondary effects that complicate treatment, especially weight gain. In the case presented, the patient does gain weight with Olanzapine and adherence is compromised. In these cases, professionals try to look for alternatives, either to try another drug or use adjunct treatment. In this patient, a change to lithium was made. In the last few years, adjunct treatment has gained traction, like for example: metformin and topiramate. The latest discovery in this matter are opioid antagonists: single dose oral tablet Olanzapine/samidorphan.

**Conclusions:**

Even though Olanzapine is one of the most effective antipsychotics and medications in mental health, its impact on patients’ weight hinders treatment continuity. The use of other already known medications and the appearance of new ones, that reduce weight gain probability, are the possible ways forward.

**Disclosure of Interest:**

None Declared

